# The WPA programme on implementing alternatives to coercion

**DOI:** 10.1192/j.eurpsy.2021.96

**Published:** 2021-08-13

**Authors:** H. Herrman

**Affiliations:** Centre For Youth Mental Health, Orygen and University of Melbourne, Parkville, Australia

## Abstract

**Abstract Body:**

The call for alternatives to coercion in mental health care is growing both within the profession and among people with lived experience of coercion in mental healthcare. There is widespread agreement that coercive practices are over-used. Considerable work is warranted across the mental health sector and in communities and governments to ensure that people living with mental disorders and psychosocial disabilities uniformly have access to high-quality care and support that meet their needs and respect their personhood and human rights. The question of whether coercive interventions can ever be justified as part of mental health treatment, to protect rights holders’ own interests or on other grounds, is highly contested. WPA issued a Position Statement and Call to Action in 2020: Implementing Alternatives to Coercion: A Key Component of Improving Mental Health Care after extensive consultation and review. The purpose is (1) to recognize the substantive role of psychiatry in implementing alternatives to coercion in mental health care and (2) to support action in this regard, essential to improving mental health treatment and care in all countries. The Statement recognises the diversity of views and experiences among mental health professionals, people with lived experience and their families and carers. This initial step is the beginning of a longer-term process, which requires continued engagement with WPA member societies, people with lived experience, families and other partners to encourage and support the implementation of alternatives to coercion in mental health care. https://www.wpanet.org/alternatives-to-coercion

**Disclosure:**

No significant relationships.

